# Integrated community case management in a peri-urban setting: a qualitative evaluation in Wakiso District, Uganda

**DOI:** 10.1186/s12913-017-2723-0

**Published:** 2017-11-28

**Authors:** Robin Altaras, Mark Montague, Kirstie Graham, Clare E. Strachan, Laura Senyonjo, Rebecca King, Helen Counihan, Denis Mubiru, Karin Källander, Sylvia Meek, James Tibenderana

**Affiliations:** 1 Malaria Consortium Uganda, Plot 25 Upper Naguru East Road, P.O. Box 8045, Kampala, Uganda; 2 Malaria Consortium, Development House, 56-64 Leonard Street, London, EC2R 4LT UK; 3 London School of Hygiene and Tropical Medicine, Keppel St, London, WC1E 7HT UK; 4 Nuffield Centre for International Health and Development, Leeds Institute of Health Sciences, University of Leeds, Leeds, UK; 5 Karolinska Institutet, Tomtebodavägen 18A, 17177 Stockholm, Sweden; 6 Malaria Consortium Africa, Plot 25 Upper Naguru East Road, P.O. Box 8045, Kampala, Uganda

**Keywords:** Child health, Integrated community case management (iCCM), Peri-urban health care, Health care access, Community health worker, Malaria, Pneumonia, Diarrhoea, COMDIS-HSD, Uganda

## Abstract

**Background:**

Integrated community case management (iCCM) strategies aim to reach poor communities by providing timely access to treatment for malaria, pneumonia and diarrhoea for children under 5 years of age. Community health workers, known as Village Health Teams (VHTs) in Uganda, have been shown to be effective in hard-to-reach, underserved areas, but there is little evidence to support iCCM as an appropriate strategy in non-rural contexts. This study aimed to inform future iCCM implementation by exploring caregiver and VHT member perceptions of the value and effectiveness of iCCM in peri-urban settings in Uganda.

**Methods:**

A qualitative evaluation was conducted in seven villages in Wakiso district, a rapidly urbanising area in central Uganda. Villages were purposively selected, spanning a range of peri-urban settlements experiencing rapid population change. In each village, rapid appraisal activities were undertaken separately with purposively selected caregivers (*n* = 85) and all iCCM-trained VHT members (*n* = 14), providing platforms for group discussions. Fifteen key informant interviews were also conducted with community leaders and VHT members. Thematic analysis was based on the ‘Health Access Livelihoods Framework’.

**Results:**

iCCM was perceived to facilitate timely treatment access and improve child health in peri-urban settings, often supplanting private clinics and traditional healers as first point of care. Relative to other health service providers, caregivers valued VHTs’ free, proximal services, caring attitudes, perceived treatment quality, perceived competency and protocol use, and follow-up and referral services. VHT effectiveness was perceived to be restricted by inadequate diagnostics, limited newborn care, drug stockouts and VHT member absence – factors which drove utilisation of alternative providers. Low community engagement in VHT selection, lack of referral transport and poor availability of referral services also diminished perceived effectiveness. The iCCM strategy was widely perceived to result in economic savings and other livelihood benefits.

**Conclusions:**

In peri-urban areas, iCCM was perceived as an effective, well-utilised strategy, reflecting both VHT attributes and gaps in existing health services. Depending on health system resources and organisation, iCCM may be a useful transitional service delivery approach. Implementation in peri-urban areas should consider tailored community engagement strategies, adapted selection criteria, and assessment of population density to ensure sufficient coverage.

**Electronic supplementary material:**

The online version of this article (doi:10.1186/s12913-017-2723-0) contains supplementary material, which is available to authorized users.

## Background

Integrated community case management (iCCM) programmes aim to reach poor and underserved communities by providing access to life-saving treatment where those who need it most live [1]. In the iCCM model, trained community health workers (CHWs) are typically equipped to assess and treat the leading infectious causes of child mortality (malaria, pneumonia and diarrhoea) in children under 5 years of age [2], advise on basic newborn care, and identify and refer those in need of immediate care to a health facility. As designed, iCCM implementation strategies have primarily targeted hard to reach, rural areas, which tend to be underserved by facility-based care [1]. In these contexts, evidence from a number of countries has now shown iCCM to be an effective and feasible strategy for improving equitable access to treatment [3–9].

There is little evidence to support the implementation of iCCM as an appropriate public health strategy in non-rural contexts or to identify any differences in implementation strategies that might be required. Mixed results from rural-urban comparative studies of community case management interventions for malaria and pneumonia have been reported, with treatment coverage by CHWs in urban areas ranging from less than 1% to an average of 40%, and consistently lower uptake of CHW services observed in urban areas than in rural [10–13]. Lower uptake in urban areas has been attributed in part to low awareness or low acceptability of CHW services, or a preference for health centres [10]. It appears a few countries have also introduced iCCM or other community case management programmes (for pneumonia or malaria) in peri-urban areas [14], however this has not been well-documented. In some cases, the targeting of peri-urban areas likely reflects horizontal equity approaches (regional or national health system coverage imperatives) or ambiguity in demarcating target areas due to the rapid transformation of once rural zones. Although community-based delivery strategies have been effectively implemented for preventive health, outreach and referral interventions in peri-urban settings [15–18], it seems there has been no explicit study exploring whether iCCM is an appropriate strategy for meeting the treatment needs of peri-urban communities.

The term “peri-urban” is frequently used, but inconsistently defined [19]. In Uganda and other sub-Saharan African countries, peri-urban zones typically feature as dynamic, transitional environments arising from the interaction between urban expansion and surrounding rural areas, where the speed of population increase is high due to migration from cities [20]. Treatment needs may not be effectively met by either urban or rural models of health service provision, and may not be catered for as peri-urban settlements often arise outside of official urban planning [19, 21]. Compared to rural areas, peri-urban communities often benefit from a greater number and diversity of proximal health services as a result of higher population densities and competition in the health care marketplace. In particular, the private sector features as an important source of child health care (in both rural and urban areas) in Uganda and other countries [22, 23]. However, the poorest community members may still experience difficulties in accessing timely treatment due to a range of common barriers which persist across rural, peri-urban and urban areas: the cost of accessing services, availability of skilled personnel or drugs, provider acceptability, quality of care, and difficulty mobilising resources once the decision to seek outside care is made [24, 25]. In peri-urban settings, these barriers may be compounded by issues of social exclusion and the breakdown or absence of social networks in transitional environments. Moreover, in areas of rapid population growth, already overburdened public health systems may struggle to keep pace with the burgeoning demand for services. Recent evidence from Uganda and other countries has suggested that CHWs can be effective in reducing the treatment burden at health facilities, as well as catering for unmet demand [26–30].

Building on prior country experience with home-based management of fever and other community-based programmes, Uganda introduced a new, comprehensive CHW strategy in 2010 – based on village health teams (VHTs) – aimed at improving health care provision, accessibility and utilisation, and guided by three principles: community ownership; equity and access; and community support [31]. The VHT is fully integrated into the health system, and is considered to equate to a ‘health centre I’, or the lowest tier in the system. The VHT model provides a platform for providing a basic package of health promotion, behaviour change and preventive interventions, with an add-on component of child treatment interventions via iCCM. VHTs typically comprise five volunteers per village, selected by the community (guided by set criteria defined by the Ministry of Health) to serve the people in their areas of residence. Two VHT members are selected for iCCM training, a 6-day, modular course in assessing and treating sick children aged 2 months to 5 years, guided by the use of a pictorial ‘sick child’ job aid [32]. This includes assessment of cough/pneumonia, diarrhoea, fever/malaria and danger signs; administration of pre-referral treatment and referral; advice on routine newborn preventive care; and screening for acute malnutrition. A box of appropriately dosed and packaged medicines, registers and other essential commodities is provided to each iCCM VHT member, with planned quarterly restocking. In practice, iCCM restocking delays have occurred and remain a persistent challenge [33]. Health facility workers provide technical supervision to VHT members through home visits and quarterly meetings. All iCCM services are provided free-of-charge, as are services at referral health centres. However, it is important to note that referred patients are sometimes required to purchase medicines and other commodities from the private sector due to recurrent stockouts in the public health system.

Uganda’s VHT strategy was designed to bridge the health human resource gap primarily in rural areas where the majority of people live and where access to health services is limited [31]. However, iCCM has also been introduced in some peri-urban areas, larger towns and trading centres as funding has allowed, in line with a horizontal equity approach (district and regional coverage) and in recognition of broader geographic needs to improve access to quality care for common childhood infections if national targets for reducing child mortality are to be reached. Despite key differences in context, Uganda’s iCCM implementation guidelines are the same irrespective of setting and there has been little assessment of implementation experience in peri-urban areas to explore suitability, with the overall aim of effective resource prioritization on a regional or national level. This study aimed to inform future iCCM implementation strategies by giving insight into caregiver and VHT member perceptions of the value and effectiveness of iCCM in peri-urban settings in Uganda.

## Methods

### Study setting

Data collection was conducted in seven villages in Wakiso district, a rapidly urbanising area surrounding most of the Kampala metropolitan zone (district headquarters are located roughly 20 km from the capital city), situated in the Central region of Uganda. With more than 2 million inhabitants, Wakiso is the most populated district in Uganda according to 2014 census estimates and has nearly doubled in size from 2010 projections [34, 35]. Six highways and roads transect the district, connecting regional urban centres to the capital. The study was embedded within ongoing iCCM implementation in eight districts in the Central region, which was supported by a non-governmental organisation (NGO) in collaboration with the Uganda Ministry of Health and UNICEF. Implementation followed the national VHT strategy and operational guidelines [31] and iCCM implementation guidelines [32], with routine iCCM service provision in the study district beginning in March 2011.

### Study design

This qualitative evaluation used participatory methods to explore caregiver and VHT member perceptions of the value and effectiveness of iCCM in peri-urban settings. The initial intention was to conduct a comparative evaluation exploring key differences in iCCM implementation between rural and peri-urban settings, given that the Central region of Uganda appeared to offer a context well-suited to exploring potential variations. However, due to the rapid urbanisation of the district and complexities in village classification described below, data were in fact collected in a range of peri-urban settlements.

The study design and scope of enquiry drew on Obrist et al.’s ‘Health Access Livelihoods Framework’, which incorporates health service and health-seeking approaches and situates access to health care in the broader context of livelihood insecurity [24]. The use of this interdisciplinary framework as a basis for evaluation aimed to facilitate exploration of the unique interplay between iCCM and the particularities of a dynamic, peri-urban context. Obrist et al. define five dimensions of access which influence the course of the health-seeking process (availability, accessibility, affordability, adequacy and acceptability), and which can serve as useful criteria for exploring user and service provider perceptions of the value and effectiveness of iCCM (Table 1).

**Table 1:** Main domains of exploration (based on Obrist et al. [24])^a^

Dimension of access (defined by Obrist et al.)	Key questions adapted in relation to iCCM services
Availability The existing health services and goods meet clients’ needs.	• What services do iCCM VHT members provide? Are the types of services provided by VHT members perceived to be appropriate? (What should VHT members be allowed to do or not do? Do iCCM services and goods meet caregivers’ needs?) • Are there sufficient numbers of iCCM VHT members available to perform services? Are they able to meet demand? • Are iCCM treatment and commodities available? Is supply sufficient to meet demand? Are the types of commodities available sufficient and appropriate to the services provided?
Accessibility The location of supply is in line with the location of clients.	• Is the location of iCCM VHT members in line with the location of the community? • Are iCCM services easily accessible via VHT members? (Are they proximally located? Is transport required? Time required to reach?) • Are services at the referral health facility easily accessible (distance, time and transport required to reach)?
Affordability The prices of services fit the clients’ income and ability to pay.	• What are the direct and indirect costs associated with accessing iCCM services? • What are the direct and indirect costs (transportation, lost time and income, fees) associated with accessing referral services?
Adequacy The organization of health care meets the clients’ expectations.	• Does the organisation of iCCM services meet community expectations? (i.e. how services are provided) • Is there adequate space for service provision? • Are the VHT members’ homes and treatment areas clean and well-kept? • Are supplies and other materials well organised? Is the organisational set-up adequate? • Do the opening hours (availability of VHT members for service provision) match caregiver needs? Can the VHT members be easily located when needed?
Acceptability The characteristics of providers match with those of the clients.	• Do the characteristics of iCCM service providers match with the expectations of the community? Do caregivers feel welcome and cared for? • How do the characteristics (attitudinal and performance) or personality of VHT members influence iCCM acceptability? • How do perceptions of VHT member competency or skills influence iCCM acceptability? (Are VHT members perceived to be qualified to provide services? Do caregivers trust the competency of VHT members?) • What is the acceptability of the available iCCM treatments and information provided? • What was the acceptability of the process through which VHT members were selected? Was the community sufficiently engaged? How did VHT selection influence VHT member acceptability?

^a^Adapted from Obrist et al. ‘Five Dimensions of Access to Health Care Services’ in the ‘Health Access Livelihoods Framework’ [24]

This study used both traditional qualitative methods (key informant interviews), as well as a series of “visualisation” tools, techniques that are commonly used in rapid appraisals to elicit information for action [36, 37], and which served as platforms for generating group discussions (Table 2). In each village, these activities were undertaken with a group of community members and, separately, with the two iCCM VHT members over a period of multiple days. The range of activities was designed to provide participants with the opportunity for a detailed, purposeful examination of a wide scope of evaluation criteria related to how well iCCM services matched communities’ child health seeking needs (derived from Obrist et al.’s dimensions of health care access), while situating iCCM within the larger health care and socioeconomic context found in peri-urban settings [24]. This participatory approach was also pursued in line with the ethos of the VHT strategy which stipulates that ‘the community is responsible for selection, supervision and support of the VHT. The VHTs are fully accountable to the communities in which they operate and their services/responsibilities are community driven’ [31].

**Table 2:** Data collection methods and tools

Target groups per village	Data collection activities
	*Rapid appraisal activities (visualisation tools to generate group discussions)*
One group of caregivers and one group of iCCM-trained VHT members (Activities conducted separately with each group)	Transect walk: conducted by participants, with guidance from the moderator. Observed village boundaries, locations and basic characteristics of all health service providers, and other important landmarks so as to acquaint the study team with the village and inform subsequent activities.
	Health service venn diagram: identified all available health service providers and organisations within and beyond the village, including external sources of power or influence; explored the relative importance of various actors; mapped relationships that influence health service delivery and uptake
	Historical matrices: explored changes pre and post-iCCM introduction in demographic factors, livelihood assets (human, financial, natural and physical capital) and available health services, gathering a detailed description of the dynamic, transitional nature of peri-urban environments
	Health service delivery matrices: explored relationships influencing service delivery and uptake (covering iCCM implementation, process, results), gathering perceptions of various trends over time related to health service delivery and uptake
	Problem and solution ranking: participants first identified and prioritised problems related to iCCM, and then discussed and ranked potential interventions to address the identified problems
	*Key informant interviews*
Community leaders	Historical profile: in depth interviews conducted with community leader or long-term resident exploring present village characteristics, historical information on the village (demographic, household, economic, livelihood assets, transport), available health care services and care seeking practices
Non-iCCM VHT members	Semi-structured interview: topics covered VHT member role, selection, training, working relationship with iCCM VHT members, and areas for improvement

### Participants and sampling

The initial sampling frame included all villages (Local Council 1 s [LC1s]) in Wakiso district that were targeted for the first round of iCCM training (March 2011), so as to maximise the period of implementation for evaluation and minimise any iCCM implementation differences across study villages. Villages had been previously classified as ‘rural’, ‘peri-urban’ or ‘urban’ by the Uganda Bureau of Statistics, based primarily on administrative boundaries. However, there is no operational definition for peri-urban in the Uganda context. We therefore sought to validate potential study villages using the decision tree proposed by Makita et al. for urbanicity classification of LC1s in the Kampala area [20]. Makita’s model incorporates the proportion of the population engaged in full-time farming, the speed of population increase, migration patterns, and the availability of space for crop cultivation, with the key indicator of peri-urbanicity defined as “rapid population change due to migration from the city by house construction” [20]. To avoid inclusion of urban areas, trading centres and communities no longer retaining space for crop cultivation were excluded. Nine potential study villages were visited by the research team, and a final subset of seven villages, including some distinctly peri-urban (close to the outskirts of Kampala) and some more ‘rural’ villages (the majority of the population was still engaged in farming), was selected. However, during data collection and analysis, it became apparent that the more ‘rural’ study villages featured some ‘peri-urban’ characteristics, and that the villages were similar with regards to health services and transport facilities. The number of villages selected was estimated based on expectations for data saturation, also considering available time and budget.

In each of the seven villages, all LC1 Chairpersons (community leaders) and non-iCCM VHT members were approached for KIIs, and all iCCM VHT members were asked to participate in the rapid appraisal activities. In each village, approximately 12 caregivers of children under five who had been resident in the village for a minimum of 2 years and had previously utilised VHT services were approached by iCCM VHT members. Caregivers were purposively selected to include a range of age, gender, and socio-economic situations; final selection was validated by a member of the research team prior to participation in the study. A total of 85 caregivers and 14 VHT members participated in the rapid appraisal activities (Table 3).

**Table 3:** Summary of study participants

Village	Rapid appraisal activities/group discussions		Key informant interviews	
	Caregivers	iCCM VHT members	Community leaders	Non-iCCM VHT members
Village 1	12	2	1	2
Village 2^a^	12	2	n/a	n/a
Village 3	12	2	1	2
Village 4	12	2	1	2
Village 5	12	2	1	2
Village 6^a^	12	2	n/a	n/a
Village 7	13	2	1	2
Total	85	14	5	10

^a^Key informant interviews were not completed in two villages due to logistical constraints

### Data collection

Data were collected from March to May 2012, approximately 1 year following the full-scale implementation of iCCM in the study area, allowing sufficient opportunity for community members to utilise VHT services and form opinions about the value and effectiveness of iCCM in their communities. KII guides and field toolkits comprising the rapid appraisal activities were developed and pre-tested in one of the seven villages; these data were included in the analysis as resultant changes to the study tools were not considered significant. Data collection took place over 5 days in each village. Fifteen KIIs were conducted in five of the seven villages (KIIs were not conducted in two villages due to logistical constraints). Activities were conducted at a suitable setting within the community (school, community hall, church, or community leader’s house) and interviews took place primarily at the respondent’s home. In each village, data were collected by a team of four external research assistants (two male and two female) who had prior experience in collecting qualitative data and were recruited with support from a lecturer at Makerere University. Research assistants participated in 4 days of training and were briefed on iCCM implementation, but were not involved in any aspects of health service delivery. Fieldwork was overseen by MM. Interviews and discussions were conducted in Luganda, and were audio-recorded in addition to hand-recorded field notes, which were reviewed and expanded at the end of each day. Every 2 weeks, data were fully translated and transcribed verbatim by the local researchers from the audio recordings, with use of field notes to cross-reference. Following the completion of data collection in each village, a feedback session was held with the research team, study participants, VHT members and community leaders, providing a final opportunity for review and validation of findings.

### Analysis

Thematic analysis followed the ‘Framework’ approach [38, 39]. The initial coding frame was based on the scope of enquiry and evaluation criteria derived from the ‘Health Access Livelihoods Framework’, and was revised and expanded as new categories or codes emerged from the data (Additional file 1). Data were primarily coded by RA, with regular input, review and discussion with KG to reach consensus on final codes. The codebook was also periodically reviewed by RK. Statements were dual coded where relevant to identify more than one idea, as well as to capture relationships between categories (for example, the perceived impact of a particular characteristic of VHT services). Themes were first summarised for each group of participants (caregivers and VHT members) in each village, and then compared across villages to explore the most prominent themes, triangulate caregiver and VHT member perspectives, and subsequently to explore any potential context-specific variations associated with the degree of peri-urbanisation. A preliminary thematic summary was prepared for each of the evaluation criteria, in alignment with the ‘Health Access Livelihoods Framework’ (Additional file 2). Given the detailed scope of these criteria, themes were then further grouped and summarised under broader categories related to the perceived value and effectiveness of iCCM in peri-urban settings, as presented in the findings below. RK, HC, KK and CS reviewed the preliminary thematic summary.

## Results

### Study participant and village characteristics

The majority of caregiver participants were female (89%), with one to two male participants included in six of the seven villages. Self-reported literacy for caregiver participants was 92%, ranging from 58 to 100% for each caregiver group/village. The majority of iCCM VHT participants were also female (71%).

Community leaders estimated village population sizes as ranging from approximately 500 to 8000, although there was uncertainty given the rapidity of change. Population growth due to urban expansion was reported in all villages, with migrants increasingly drawn by land purchase, new house construction, the availability of rental rooms, casual labour and educational opportunities. Population growth was also reported to be fuelled by natural increase, reflecting both high birth rates as well as decreased death rates, which respondents associated with improved access to prevention and treatment.

Participants from all villages described a dynamic economic situation in the district, noting the rapid pace of land sale, reduced space for agriculture and an overall decline in farming. By definition, these land tenure changes appeared to be more extensive in the highly peri-urban villages, whereas those villages with still some rural characteristics reported more recently encountering displacement and occupational change. Business was the main source of income reported in three villages, while a mix of agriculture, animal husbandry and casual labour continued to predominate in four villages. The more agricultural villages in the study reported having less financial capital, with lower household incomes, and more limited access to cash resources and credit.

*The village has developed so there is no land for agriculture. Most land is being taken on for settlement and other business. In the future, this land is going to become smaller. (Caregiver, Village 5)*

*Mainly people have small businesses – especially the women – like kiosks, retail shops, boda boda (motorcycle taxi) riders, brickmaking, bars, some people can sell water if one has got a big tank. (Community leader key informant, Village 6)*

At least one school was located in each village and a majority (ranging from 60 to 90%) of the population was estimated to have ever attended school. Although there were some differences in access to transport facilities, *boda bodas* and bicycles were widely reported to be available when required, with an increasing number of cars reported in the most peri-urban villages.

All but one village had a private clinic located within the village, traditional healers featured in all villages, and NGO or church-operated health services were available in two villages. Study villages reported utilising or linking with three to seven health service providers outside the village; these included a range of public health centres and hospitals, private clinics and hospitals, drug shops, traditional healers and a few NGOs. Some participants observed that a growth in health services had followed the recent population increase. However, increased demand for services was also perceived to have resulted in health facility congestion in some areas.

### Effective treatment access

As designed, iCCM services were universally perceived to be effective in facilitating timely treatment access, particularly by low income earners, who might otherwise be forced to delay initial care seeking while mobilising cash resources for transport or treatment. However, perceptions of effective treatment access were diminished in one village where poor VHT member availability was observed. Improved treatment access (including prompt referral treatment), combined with quality care, was perceived to have led to reduced child mortality and severe disease in all villages. Caregivers also consistently observed that fewer children experienced complications requiring hospital admission or resulting in negative sequelae. Compared to the year prior to iCCM introduction, caregivers widely observed that they now had healthy children, who were energetic and bright, rarely ill and experienced proper growth.

*The LCs no longer make burial letters because the children no longer die like it was in the past. (Caregiver, Village 5)*

High utilisation of iCCM services was reported across villages, with many caregivers reportedly shifting from primarily attending private clinics and traditional healers to VHT members as a first point of care. There was also some limited mention of switching from health centres, hospitals and pharmacies as first point of care, as well as reduced self-medication or home use of herbal remedies.

*Most women have stopped taking their children to traditional healers and clinics, they take them to VHT [members]. If they fail, the children are referred to hospital. (Caregiver, Village 1)*

Relative to other health service providers, participants valued VHT members’ free, proximal, 24-h availability; caring attitudes; treatment quality; perceived competency and protocol use; unique follow-up services; and effective referral services.

#### Cost, proximity and 24-h availability

Improved treatment access was largely attributed to VHT members’ proximity (easily accessible by foot), long hours of service availability (including night-time), and free service provision. Participants valued the availability of free medicines, contrasting this with the high cost of medicine in private drug shops, which were at times the only source for ACT due to stockouts at public health facilities. Rapid access to VHT services was contrasted with the significant amounts of time required for travelling to and waiting at health facilities (sometimes leading to recurrent care seeking due to stockouts), as well as for seeking herbal remedies.

*When the child gets ill, she gets prompt treatment and gets better because treatment is free and near. Whereas for other service providers a child may spend three days with no treatment as one looks for money and it is far. (Caregiver, Village 3)*

*There are no lines to make so you just reach and then get treatment, thus no time is wasted. This has saved us from the long lines we used to make in [name] health centre. (Caregiver, Village 6)*

#### Caring attitudes and performance

VHT members were perceived to be welcoming, caring, sympathetic, calm, *‘not abusive or shouting at’* caregivers, and *‘performing their jobs with love’*, attitudes which caregivers in some villages contrasted with the rude or callous behaviour of health facility workers and private clinicians. VHT members were also largely perceived to perform quickly and responsibly; be hard working, dedicated and *‘ready to serve’*; and act with integrity, providing services equitably and not selling iCCM resources.

*The iCCM VHT [members] welcome and care for us so we can easily go back to them for health care. Unlike in other health facilities where health providers are so rude. (Caregiver, Village 7)*

#### Treatment quality and acceptability

Children were widely reported to heal quickly following VHT treatment, which was attributed to both effective, fast-acting drugs and provision of a complete course of treatment. This was most consistently reported for malaria and diarrhoea, with some mixed reports on the effectiveness of pneumonia treatment. VHT-provided medicines were also generally perceived to be of good quality, unexpired and without side effects. Caregivers in some villages emphasized a preference for VHT medicines compared to some treatments provided at private clinics and health facilities, appreciating both the quality and ease of administration of VHT medicines. In particular, participants frequently contrasted the acceptability of VHT-provided tablets with the common use of injections, which were associated with adverse effects and negative sequelae, as well as the prescription of syrups, which were sometimes perceived to be ineffective.

*In the past the children used to fall sick and go to the private health providers who sometimes do not have the skill and this comes with its own complications. Like here in our village we have two people who were affected because of the injections. (Caregiver, Village 5)*

*The medicine they give is so effective and in three days the child is better. Previously we could buy syrups which were not effective at all. (Caregiver, Village 3)*

#### Competency and protocol use

Caregivers largely perceived that VHT members were qualified, trained, skilled, and knowledgeable, expressing confidence in VHT member competency in nearly all villages. This confidence was fostered through effective treatment, referral and follow-up experiences, underpinned by trust of a known person in the community. Participants also widely recognised provider use of iCCM protocols, frequently observing that VHT members acknowledged their limited capacities, followed strict guidelines, or used tools (job aids, diagnostic tests) to determine treatment and referral. VHT members also reported that they showed the job aid to caregivers, thus instilling confidence that they were performing according to training. Caregivers appreciated that VHT members referred immediately and “did not insist” on treating illnesses they could not manage, unlike “business-oriented” private clinics.

*In [private] clinics, even when they find that they will not treat the patient, because they are business-oriented, they continue to give you medicine which will not heal the person. Whereas the VHT [members] refer you immediately, before the child gets seriously ill. (Caregiver, Village 7)*

*The iCCM VHT [members] do have cards they use to administer medication. They also first thoroughly check the patient before administering medication because they have the testers to use. (Caregiver, Village 6)*

*We treat as the caregivers read the job aid too, because it’s written in Luganda. So they know that we don’t just think about the treatment, but we do what we are supposed to do. … We follow the job aid and beyond that, we don’t do anything other than referring the patient. (VHT member, Village 5)*

#### Unique follow-up services

In contrast to other providers, VHT follow-up services were perceived to enhance quality of care, facilitating correct treatment adherence through careful explanations and verification of correct treatment administration. In addition, follow-up visits were perceived to promote caregiver capacities, hygiene and sanitation, and other healthy behaviours, with caregivers frequently emphasising that they were encouraged to maintain good practices in their homes because of the potential for VHT member follow-up. Participants in some villages underlined that follow-up services distinguished VHT services from private clinics and alternative service providers, enhancing VHT acceptability and utilisation.

*They always come to do follow up to ensure that the children are fine after the treatment they give to us. Some parents are lazy when it comes to giving their children medicine so they ensure to come to us and make sure we have given our children medicine in full dosage. (Caregiver, Village 2)*

*[In private clinics] it’s the caretaker to take back the child and seek advice. The VHT [members] do follow-ups and in case the child does not get better, he gives you a referral. (Caregiver, Village 1)*

#### Referral system

Caregivers in all villages emphasised the pertinence of iCCM referrals and VHT members reported that caregivers ‘respected’ referrals when the VHT member could not manage the illness, even if they preferred to avoid it, given the intrinsic difficulties associated with referral care seeking. Participants also widely observed that referral helped VHT members to avoid *‘death on their hands’*, underscoring that it was not only potentially life-saving, but essential for upholding VHT members’ reputations and continued acceptability as service providers. A high value was attached to the referral letter, which was perceived to enable prompt treatment *(‘saving lives’*) and reduce time spent at the health facility. Patients with referral forms were generally not required to queue and health facility workers could rapidly obtain pertinent information from the form. VHT members also sometimes facilitated referral by helping to organise transport or accompany the patient/caregiver to the referral facility to expedite receipt of care. Privileging patients referred from VHT members also served to reinforce perceptions of VHT member competency and subsequent utilisation.

*Caretakers too have believed in us in that when we refer them, they now accept, because they get different treatment from there, unlike before when they would insist by saying, ‘Do what you can for me.’…The patients used to plead with us to do anything in line of treatment, but today they do not plead, they just go. (VHT member, Village 2)*

*When they treat and see that the child is not getting well, they immediately refer. They also escort us during referral, and at the health centre, they deal with us immediately. We don’t have to make long lines. (Caregiver, Village 4)*

### Factors limiting VHT members’ perceived effectiveness

Despite overall high reported utilisation of VHT services, key gaps in the availability of diagnostics, newborn care and drugs were widely reported and perceived to result in referral or necessitate use of other health service providers. This alternative care seeking was, in turn, perceived to result in direct and indirect health seeking costs, delayed treatment access, and increased illness severity or mortality. VHT member absence and low community engagement in VHT selection were also perceived to limit effectiveness in some villages. Although the referral system was valued, caregivers’ ability to access referral treatment was limited by transport barriers and gaps in referral centre services, problems that were accentuated by perceptions that many referrals might have been avoided through improvements in VHT service availability.

#### Types of services (diagnostics, newborn care)

Both caregivers and VHT members cited a growing demand and expectation for ‘blood testing’ and other diagnostic services. (In this setting, VHT members did not have malaria rapid diagnostic tests.) Respiratory timers were utilised to assess for fast breathing, however these were sometimes perceived to be of poor quality or frequently broken, and “more sophisticated” tools were requested.

*We know that anyone with malaria has to be measured with thermometer, so we don’t feel comfortable if it’s not used. (Caregiver, Village 4)*

*Some people just prefer going there [to the health centre I] because for them, they can even do blood testing for the children which we don’t do as VHT [members] since we don’t have the machines. (VHT member, Village 7)*

The need for additional training, medicine and equipment to manage newborns was underlined by caregivers and most VHT members across villages, with some suggesting that this would reduce mortality and referrals. However, in one village, VHT members underscored the challenges and risk of mortality associated with managing newborns, noting that they would prefer to restrict their involvement.

*Most times after birth the mothers are weak and cannot easily move and yet if the iCCM VHT [members] had the skills this would be solved. (Caregiver, Village 5)*

There were also a few VHT member reports that poor acceptability of the absence of services for older children drove low utilisation by some caregivers.

#### Drug stockouts

Poor availability of medicines was ranked as a top problem in six villages. Drug shortages were perceived to result from high demand (poor quantification), lack of responsiveness to stockout reports, and absence of transport to collect commodities from the health facility. In three villages, some respondents also associated the poor availability of medicines with the limited number of VHT members. The lack of diagnostics and persistent drug stockouts were perceived to reduce acceptability of and confidence in iCCM services, contributing to diminished caregiver and VHT member morale and sometimes discouraging return use.

*Most times the medicine is not there for pneumonia. (Caregiver, Village 5)*

*The medicine given takes two months and we spend the third month with no medicine because they restock after three months. (VHT member, Village 6)*

#### VHT availability

Although in most villages VHT members were reported to offer long hours of service availability, there were reports of occasional absence due to other activities, with some caregiver acknowledgement that the service was voluntary work and that VHT members had other responsibilities. VHT member absence was reported to occasionally lead to utilisation of private clinics or health facilities and there were wide reports of VHT member absence in two villages; in one community, a VHT member was never available during the day due to outside employment.

*When it comes to the time they offer the services to us, we cannot be strict on this because it is voluntary work. They open the services to us at their convenience, but they endeavour that they give to us the biggest portion of time. (Caregiver, Village 2)*

*We can walk like four times climbing that hill to look for the VHT [member]. I get tired yet I have my small business and the child is getting worse, I get so tired that I can’t do other work and I have not got the medicine… In most cases she is not at home. [She] goes at 7:00 am up to 6:30 pm and the child is worsening. You cannot stay home waiting for referral letter. Shall we move at night? (Caregiver, Village 3)*

Caregivers also generally perceived that two iCCM VHT members were insufficient to meet the high demand, given the large population size. However, these perceptions also reflected some cases of poor distribution of services, due to large geographic areas or inappropriate VHT member selection as described below.

*The population is high; it cannot be served by two VHT [members] and we expect the population to increase. (Caregiver, Village 6)*

#### Low community engagement

In the most urbanised study villages, low community engagement in iCCM VHT selection was perceived to result in poor distribution of iCCM VHT members; diminished awareness and ownership of services; and, in one village, the selection of a VHT member with political engagements. Across villages, there were perceptions of inadequate involvement or transparency in the second stage of VHT selection, which involved identifying two of the five VHT members for iCCM training and service provision. Some suggested this was determined by health workers or other authorities, and did not consider the geographic distribution of the iCCM VHT members. Although perceptions of inequitable geographic coverage did not appear to have an important effect on overall reported iCCM utilisation, participants observed that some community members, who were generally located on the village periphery, experienced inequitable access, limited benefits and lost time in seeking VHT services. Politicisation of services in one village was perceived to result in service discrimination and appeared to hamper overall caregiver satisfaction and utilisation. Caregivers in villages reporting low involvement attributed this to poor awareness at the time of program initiation and lack of opportunity to participate, possibly reflecting diminished social cohesion, inadequate administrative structures or unrepresentative local governance in peri-urban areas which may hinder community engagement. By comparison, in the more agricultural villages, participants consistently reported higher community involvement in VHT selection. Participants attributed this to an active local council chairman, effective sensitization, and community receptiveness to the programme, observing that this ultimately enhanced VHT members’ acceptability and accessibility due to the appropriate location of services.

*They just planned this program for us and we were not involved at all. The VHT [members] stay in their homes and operate their work from their homes. We can’t decide therefore where they operate their work from. (Caregiver, Village 5)*

*They are not located properly. Because they are few they are not distributed well in the village. Some beneficiaries travel long distances to get to their homes to receive treatment. (Caregiver, Village 7)*

#### Non-use of VHT services

Those who could afford private doctors or clinics were reported to privilege these, while others who were reported to opt for alternative health service providers were influenced by doubts regarding VHT competency or drug quality. Although there was some indication that trust had increased over time, there were sporadic comments identifying community members, primarily wealthy and educated people, who did not trust VHT qualifications or felt their training was insufficient and thus did not utilise VHT services. Also, there was limited mention of some community members who did not trust the quality or effectiveness of iCCM medicines, in some cases believing the programme to be politically motivated. It was suggested that training certificates or other formal documents would be useful for attenuating doubts and fostering acceptability among community members more accustomed to seeking proof of qualifications in a diverse health care marketplace. Moreover, relatively wealthy community members, who are not targeted under the iCCM strategy, were reported to be less aware of iCCM services, particularly in the highly peri-urban villages, where participants reported there was slightly less universal awareness and knowledge of VHTs and iCCM services (likely a proxy for the lower overall community engagement reported in these villages).

*The rich people are not comfortable with the iCCM VHTs’ service. The very educated first want to see someone’s documents papers before accessing services. They even don’t see them with uniforms. They believe in someone with documents. They say, ‘What kind of service someone can provide from home?’ (Caregiver, Village 5)*

*In our village, you may know someone as a farmer or businessman, but when you are told that is currently giving children drugs you cannot accept it easily, so you decide to go to the hospital. It is not easy to trust the VHTs; we are not sure of their training. (Caregiver, Village 7)*

#### Referral gaps

Despite the reported access to transport facilities across villages, lack of transport to the referral health centre was ranked as a major problem in six villages, inhibiting or delaying referral completion. Accessing referral and facility-based newborn care often was perceived to be costly, sometimes prohibitively so, involving both loss of time and transport-related expenditures as well as direct costs at the referral facility. Although referral services were intended to be free of charge, costs were incurred for drugs, medical supplies and other costs associated with hospital admission, largely due to stockouts or inadequate resources at the referral facility. Moreover, gaps in referral service availability (stockouts, health workers unavailable at night) were reported to lead to recurrent referral elsewhere, prolonging the care seeking journey and delaying treatment access – events and costs which caregivers often perceived could have been avoided had sufficient resources been available at the VHT level. Diminished acceptability of referral in five villages was largely driven by these perceived limitations in iCCM service availability (limited newborn care, drug stockouts, absence of diagnostics), compounded by the sometimes poor availability and quality of services at the referral facility. Despite overall positive perceptions of the referral system, caregivers in three villages reported that health facilities utilised the VHT as a gatekeeper, noting that if they sought care for a child under five (presumably one not requiring urgent care) at the public health centre without presenting a referral letter, they would be redirected to the VHT member, seemingly in order to reduce facility congestion. In Uganda, caregivers are encouraged to seek care from the nearest source, regardless of health system level. In one village, there appeared to be some negative views of this system, with reports that some caregivers who were either unwilling or unable to use VHT services were “forced to”. Other perceived limitations in the referral system included lack of counter-referral information and forms, and inadequate supply and low acceptability of the referral form. In two villages, it was reported that some health facility workers did not perceive it to be an “important document”.

*If the child is referred, the caregivers may not have money for transport, which would take some time to get means of transport and the fees while the sick child is worsening. (Caregiver, Village 7)*

*There are times they refer us but when we go to [name of health centre], we find that they do not have the services we need, like for instance putting a drip on a child. In the long run, you end up just buying these things. However, there are times when you do not even have the money to buy these things. (Caregiver, Village 2)*

*There are times when we refer the caregivers to [name of public hospital] but they always complain that they never find there medicine. The caregivers reach there and they are referred again to other service providers to go and get medicine. Some caregivers think that we intentionally refer them there and this has really affected our service. (VHT member, Village 1)*

### Impacts on livelihood assets

Economic savings was perceived to be an important impact of iCCM across all villages, as households no longer needed to access and spend cash resources to purchase medicine, attend private providers or utilise transport for initial care seeking. “Saving money from their husbands” was a minor extension of this theme in the most urbanised villages where lower overall community awareness of iCCM was reported. A few enterprising caregivers reported that, as their husbands were unaware of the free services, they would save funds provided for treating the sick child or apply this money for other purposes, suggesting increased financial autonomy. Savings translated to changes in household spending in all villages, with wide reports that financial resources were reallocated to pay for basic child needs such as food, clothing, bedding, school fees, and fluids for children under treatment, and, in a few villages, to develop businesses or income generating activities. In the most urbanised villages, there was also some mention of being able to spend money on food, hair and personal care for mothers. Reduced health seeking expenditures appeared to have a specific importance in these peri-urban settings: despite the reported rise in incomes and access to cash resources in some villages, the loss of agricultural space and decline in farming were also reported to have led to increased pressure on cash resources, with households now purchasing food and goods they may have previously produced or bartered, thus diminishing net improvements in resources available for health seeking.

*We are able to do savings since the services are free of charge. We no longer depend on our husbands so much when it comes to money for treatment. (Caregiver, Village 6)*

*It has improved the feeding of our children. The money we used to spend on buying medicine is now saved and we use it to buy milk, clothes and other basics we could want for home. (Caregiver, Village 6)*

*Men don’t like giving their wives money, so these women can't tell them about iCCM, so she saves the money for her hair management, to buy books and clothes for the children. So they don’t tell their husbands, women take full responsibility of children. (VHT member, Village 5)*

Participants in all villages also underscored that the regular availability, proximity and rapid provision of VHT services – along with the decreased frequency and duration of childhood illnesses as a result of effective treatment and prevention – enabled caregivers to save time and avoid other indirect costs (including transport and other travel costs) associated with treatment seeking and caring for sick children. This ‘saved time’ was perceived to translate into important economic benefits, allowing caregivers to return home quickly and attend to productive activities.

*Services are within the community so less time is used and we can do other work. We don’t have to go to [name of health centre] where one can spend the whole day. (Caregiver, Village 3)*

*We can be able to do our work though the child is sick because she’s on medication. This has helped us to work and have food in the homes. (Caregiver, Village 1)*

iCCM was also perceived to have increased human capital in the study communities, with wide reports of higher school attendance and improved access to education as a result of VHT services. Participants related this both to healthier children, leading to reduced absenteeism, and to decreased health-related expenditures enabling households to pay school fees and purchase scholastic materials. Increased caregiver knowledge and skills – such as newborn and sick child care, correct medicine use (administration, treatment completion, storage), hygiene and sanitation, net use, nutrition, and disease symptoms and causality – were also widely reported as a result of iCCM services, frequently in relation to VHT follow-up visits. VHT members also reported changes in caregiver beliefs regarding herbal treatments and witchcraft.

*Currently our nursery children can study for a whole term without falling sick. Initially they used to be infected from school, as one child infected others who were not sick. Now we quickly take them to VHT [members] to be treated. Children study and perform better. (Caregiver, Village 3)*

*We have learnt so many things from them, like giving full dosage for children, because they clearly explain to us how to do it. (Caregiver, Village 5)*

Finally, caregivers also widely perceived that iCCM was promoting a demographic shift, frequently noting that, in their view, the availability of free care, caregiver satisfaction with iCCM and having healthy children encouraged higher birth rates.

*Our children also grow up when they are healthy and energetic and this makes us happy as parents and what comes out of happiness is giving birth to other children. The happiness of a father is children in the homes. (Caregiver, Village 4)*

## Discussion

In this qualitative evaluation, iCCM was perceived to be effective in facilitating treatment access in a rapidly urbanising setting that differed from the hard to reach, rural settings traditionally targeted for iCCM implementation. These qualitative findings complement a recent evaluation of iCCM in central Uganda that was conducted under the same programme of implementation, but covering a wider geographic area, which found that iCCM increased treatment coverage for diarrhoea and fever, and health care seeking for all three diseases [26]. The ability to access proximal, effective treatment at no cost and at any hour drove overall high caregiver acceptability, utilisation and satisfaction with iCCM implementation in these settings. These findings are consistent with the predominantly positive community perceptions, acceptability and demand for iCCM services previously reported from rural areas of Uganda [29, 40, 41], although one study in Eastern Uganda reported relatively low use of community medicine distributors (precursors to VHTs) who were trained to provide treatment with artemether-lumefantrine and amoxicillin, finding private clinics to be the most common source of initial treatment, despite overall high appreciation of community-level quality of care [42]. The congruence with evidence from rural areas of Uganda would seem to suggest that caregiver and VHT perceptions of the value and effectiveness of iCCM may be driven in part by the prevailing Ugandan policy and implementation context. In other words, it may be that, regardless of important variations in village characteristics, iCCM is simply well-aligned and well-adapted to the broader Ugandan health system and country context, given the occurrence of national drug shortages, inconsistent availability of public health facility services and wide caregiver reliance on costly private sector services in both rural and more urban areas [22]. Buchner et al. found similar perceptions of the reliability of VHT services compared to formal health facilities, which might be closed, without staff, or have medicine stock-outs [40].

Although all villages in the study had existing health services located within or close to the community, caregivers privileged VHT services primarily for reasons of cost and proximity, underpinned by favourable perceptions of VHT care and treatment quality. This suggests that caregivers perceived that iCCM improved both equity and “effective access”, access to a trained and equipped health worker, as designed [43]. Although some evidence has suggested that consumers may perceive free drugs to be less effective [44, 45], in this study, participants reported that given the limited financial means of the target population, it would seem irrational to bypass free VHT services and incur either direct or indirect care seeking costs. Moreover, all study villages reported that these services were reaching poor people, who might otherwise delay or forgo care seeking. Although it can be complex to untangle the combination of elements driving health provider choice, factors such as provider acceptability and perceived quality of care have been shown to be influential in attracting patients [46], and in fostering continued acceptability and return use. High acceptability of VHT care has been previously widely reported from rural settings in Uganda, in part resulting from community selection of trusted, known individuals who are proximally located [40]. In the present study, it was also reported that trust in VHT competency and iCCM services increased over time, following effective treatment, follow-up and referral experiences. However, in contrast to the high acceptability of VHT care reported in these peri-urban settings, some CHW studies in urban contexts have reported limited acceptability, often related to negative perceptions of competency or training [10]. Although we did not directly evaluate quality of care measures, caregiver perceptions of VHT members relative to other health care providers in this study appeared to be consistent with evidence from a recent study in rural, western Uganda which suggested that perceived quality of care was higher for VHT members than for health facility workers [47]. Other evidence from rural settings in Sierra Leone has suggested that caregivers reporting poor quality of care as a barrier to facility care seeking were more likely to receive treatment from a CHW [48].

Interestingly, a recent evaluation of iCCM in Malawi suggested that geographic targeting of iCCM to hard to reach areas may warrant reconsideration and that distance has been perhaps overemphasized as the main barrier to care seeking in some areas [49]. The preference for iCCM services from among a choice of providers observed in this study suggests the need to go beyond geographic proximity, the main criterion applied in health planning [25, 43], and assess both the broader health service landscape and user preferences when determining appropriate targeting of iCCM implementation. These might include: the reliability of services (stockouts, personnel absence, out-of-hours availability); perceptions of quality and provider attitudes; and unmet demand and long wait times, suggesting a need for task shifting to lessen the burden at congested lower level health facilities, particularly as rapid population growth puts pressure on existing services. The perceived effectiveness of iCCM in this context indicates that it may be an appropriate complementary or transitional delivery strategy for extending treatment coverage in peri-urban areas where facility-based care falls short. Findings also highlight the need for parallel efforts to improve provider acceptability and quality of care at health facilities, as well as to sensitise communities on the health system and complementary roles of community and facility-based care, clarifying that VHT members are intended to serve as a first point of care rather than provide a comprehensive range of services. Over the longer term, the benefits of iCCM should both lead to and trigger improvements in facility-based delivery, eventually lessening the need for community-based care. Given limited resources and programme sustainability, further research might explore the cost-effectiveness of iCCM as a transitional strategy, and identify potential benchmarks for facility access and quality improvement that signify when the programme is no longer the most appropriate intervention for the context.

### Implementation issues in peri-urban settings

The importance of a participatory selection process for promoting acceptability and sustainability of CHW programs has been highlighted elsewhere [50–52]. To facilitate treatment access, VHT members must be proximally located and equitably distributed; available to provide services most of the time and easily located when needed; capable of performing according to training (typically formal education or literacy); and acceptable to the people they are supposed to serve. In this study, some specific challenges were observed with regards to selecting iCCM VHT members, which appeared to reflect both the nature of candidates in peri-urban settings, as well as barriers to effective community engagement. In peri-urban settings, where survival increasingly depends on paid wages, potential VHT candidates are more likely to be engaged in full time employment outside the home and more susceptible to changes in economic activity, raising the likelihood for potential time conflicts and reduced service hours. Moreover, those who meet the suggested literacy criterion may also be more likely to hold employment outside the home. The most urbanised villages in this study reported little to no involvement in VHT selection and programme initiation, and overall less universal awareness of iCCM, which is consistent with evidence from urban settings [10, 11]. This may have been due to specific challenges engaging communities featuring a wider range of socioeconomic characteristics, diminishing social cohesion and evolving sources of power or influence, particularly as administrative structures may be undergoing transformation or failing to keep pace with peri-urbanisation. The selection of VHT members with active political engagements indicates that power dynamics played a part in at least one village. All of these factors suggest the need for tailored sensitisation approaches and community engagement strategies, and potentially the need for adapted selection criteria in peri-urban settings.

Implementation in peri-urban settings may also need to specify measures for VHT density. Villages in this study represented a range of geographic and population sizes, however, no formal guidance was provided for ensuring appropriate coverage or distribution of VHT members, particularly at the second stage of selection for iCCM. Norms for ‘health centre I’ coverage were initially proposed in Uganda, with one VHT member per 25 to 30 households [53]. However, this standard was not applied in areas of high population density due to limited resources. Uganda’s VHT strategy generally allowed for the same number of iCCM VHT members per LC1 regardless of village characteristics. A recent study examining uptake and effectiveness of Kenya’s Community Health Strategy in rural, nomadic and peri-urban areas observed a similar challenge with the lack of attention to variations in population density when considering the number of households allocated to CHWs [18]. Establishing appropriate standards for VHT density and geographic coverage may help to achieve more equitable VHT distribution in larger communities, as well as – in combination with appropriate community monitoring – anticipate necessary adjustments over time in areas experiencing rapid population changes and potentially more frequent attrition. Given the challenges with identifying VHT members who are present full-time in the community highlighted above, VHT density standards for peri-urban settings might also need to reconsider expectations for VHT service hours [54]. As suggested by study participants, if VHT members are not present full-time in the community and able to provide services on demand, then more VHT members might be required. Alternatively, where identifying qualified VHT members with sufficient availability is problematic, it may be more appropriate to consider other models for community service delivery, such as training shop keepers or drug store owners in iCCM [55, 56].

This study adds to the description of peri-urban settings in relation to the health care context. The diffuse nature of peri-urbanicity and lack of consistent definitions have been acknowledged and elucidated primarily in the environmental and agricultural literature [19]. A review of the health literature suggests that peri-urban definitions are both study and context-specific, with the term referring to a wide range of processes and environments, from crowded, ‘slum-like’ settlements [15] to middle income residential areas and more rural hinterlands. Moreover, it would appear that many studies involving ‘peri-urban’ settings do not consistently describe classification criteria, making it difficult to assess the broader relevance of their findings. There is a need to further develop peri-urban typologies in relation to the health care context in order to support appropriate health policy and planning.

### Overall iCCM improvement design

Although users and VHT members reported overall positive perceptions of iCCM implementation and effectiveness in this context, a number of gaps were reported with regards to diagnostics, supply chains and referral care – challenges which have been reported in recent evaluations of iCCM in Uganda and elsewhere, and which require continued attention [33, 41, 57]. An expressed need for increased postnatal support in the community also has been previously observed in Uganda [58]. Referral system organisation should also receive continued attention in iCCM improvement design, with social and behaviour change communication targeting all referral actors. Importantly, challenges associated with organising and affording referral transport were still reported in these peri-urban communities, despite fairly wide access to transport facilities. Transport challenges have been shown to be a persistent problem in urban areas [59] as well as remote settings and should not be dismissed in peri-urban implementation strategies.

Table 4 presents an overview of the key conclusions and recommendations from this study.

**Table 4:** Key conclusions and recommendations

Theme	Conclusions and recommendations
Targeting of iCCM implementation	When determining targeting of iCCM implementation, health planners should consider factors beyond geographic proximity and assess the broader health service landscape and user preferences (such as reliability of services, perceptions of quality and unmet demand), particularly where rapid population growth has put pressure on existing services.
	Research should explore the cost-effectiveness of iCCM as a transitional strategy, and identify potential benchmarks for facility access and quality improvement that signify when the programme is no longer needed.
Peri-urban implementation	Develop tailored sensitization and community engagement approaches to facilitate community participation in the VHT selection process.
	Establish or review norms for VHT density and geographic coverage in larger communities to ensure equitable distribution, considering population changes and expectations for service hours.
	Potentially consider other delivery strategies for iCCM as an alternative to the VHT model, in high density peri-urban settings.
	Develop peri-urban typologies in relation to the health care context in order to support appropriate health policy and planning.
Overall iCCM improvement design	Continued attention is needed to improve diagnostics availability, strengthen supply chains and improve access along the referral care pathway in order to maximise the benefits of iCCM service provision.
	Explore options for increasing postnatal support in the community.
	Even where transport facilities exist, organising and affording referral transport remains challenging and needs to be addressed in implementation strategies.
	Sensitise communities on the health system and complementary roles of community and facility-based care, clarifying that VHT members are intended to serve as a first point of care rather than provide a comprehensive range of services.

### Limitations

This study focused primarily on understanding the perceptions and experiences of iCCM users and service providers, who were appropriately informed to evaluate implementation. The views of non-users, who may have perceived iCCM to be less pertinent or aligned with their needs, were not directly solicited. While non-users may have provided insightful input into the evaluation, the relative size of this group was believed to be small, given the expected high treatment coverage as reported in other studies in the Central region of Uganda [26]. Data were collected in a small sample of villages (due to the in-depth nature of the rapid appraisal activities and available resources), however, saturation was perceived to have been reached and a range of villages allowed for exploration of potential variations across peri-urban settings. Distance from formal public health facilities was not a criterion for village selection; there was variation in health centre proximity across study villages and this variation did not necessarily correlate with the degree of peri-urbanicity. As we targeted caregivers who had previously utilised services, iCCM VHT members were necessarily involved in assisting the research team to identify study participants. However, we aimed to limit potential selection bias by clear communication of the study protocol and a process of caregiver vetting and validation by the research team. In order to facilitate community assessment of a comprehensive set of evaluation criteria, the rapid appraisal activities utilised a purposeful approach, which may have limited the extent to which other topics emerged. Social desirability bias may have contributed to positive reports of VHT services – caregivers clearly valued VHT members, recognised their volunteerism, and may have been hesitant to critique VHT members’ work. The use of action research approaches was designed to minimise this potential bias, providing the opportunity and motivation for participants to raise problems and discuss solutions for strengthening services. Finally, although this evaluation focused on the iCCM package of services, caregivers and VHT members may have sometimes conflated this with other VHT-provided preventive and health promotion interventions.

## Conclusions

In the Ugandan context, both users and service providers perceived iCCM to be a pertinent, well-utilised and effective programme for improving treatment access and raising the health status of children under five in peri-urban communities, reflecting both positive perceptions of iCCM services and perceived gaps in existing formal and informal health services. Community-based delivery may be an important transitional strategy in peri-urban areas where the quality or accessibility of facility-based services is inadequate. Implementation of iCCM as an appropriate strategy in peri-urban settings depends in part on questions of national health system organisation, cost-effectiveness and the need to task shift to lessen the burden at lower level health facilities. Further research might explore its cost-effectiveness as a transitional strategy and seek to identify potential benchmarks for facility quality improvement that signify when the programme is no longer needed. Implementation in peri-urban settings should give careful attention to the initial process of establishing iCCM; tailored sensitization and community engagement approaches may be required and particular attention should be given to facilitating broad community participation in the VHT selection process, which may be more challenging in dynamic, peri-urban settings with diminished social cohesion and shifting populations. Consideration should also be given to establishing or clarifying criteria or norms for equitable VHT coverage. Other delivery strategies for iCCM, such as the training of private providers, may also be appropriate alternatives or supplements to the VHT model in high density peri-urban settings. As has been reported in evaluations of iCCM across contexts, continued attention is needed to strengthen supply chains and improve access along the referral care pathway in order to maximise the benefits of iCCM service provision. This study mapped iCCM to the Health Access Livelihood Framework, evaluating a broad scope of criteria and generating an expansive coding frame which may be useful for informing evaluation frameworks. Although the majority of people will continue to reside in rural and urban settings requiring the greatest public health attention, the unique health service needs of peri-urban communities should receive more careful consideration in health system policies and planning, and in health services research.

## Additional files

Additional file 1: Abridged Codebook. The revisions and expansions to the coding frame (initially based on the ‘Health Access Livelihood Framework’) as new categories or codes emerged from the data. (DOCX 90 kb)

Additional file 2: Summary of user and VHT member perceptions of iCCM implementation in peri-urban settings. A preliminary thematic summary was prepared for each of the evaluation criteria, in alignment with the ‘Health Access Livelihood Framework’. (DOCX 40 kb)

## Abbreviations

CHW: Community health worker
iCCM: Integrated community case management
KII: Key informant interview
LC1: Local Council 1 (local governance body for village unit)
MOH: Ministry of Health
NGO: Non-governmental organisation
VHT: Village Health Team (community health workers in the Ugandan health system)
