# Supplementary material for: Integrated community case management in a peri-urban setting: a qualitative evaluation in Wakiso District, Uganda
Source: BMC Health Serv Res. 2017 Nov 28;17:785. doi: 10.1186/s12913-017-2723-0 (PMC5706411; doi:10.1186/s12913-017-2723-0)
Supplement: Supplementary file 1 — Abridged Codebook. The revisions and expansions to the coding frame (initially based on the ‘Health Access Livelihood Framework’) as new categories or codes emerged from the data. (DOCX 90 kb) [file 12913_2017_2723_MOESM1_ESM.docx]

**Additional File 1. Abridged codebook**

*Blue shading Indicates code related to provision of services or impact on provider*

| **Category** | **Abbrev. Category** | **Definition** | **Code** | **Abbrev. Code** | **Description** |
| --- | --- | --- | --- | --- | --- |
| **Availability** |  | **Existing iCCM services and goods meet community child health needs** | | | |
| Availability - iCCM services | AVAILSER | Types of services provided by VHTs (perceptions of what VHTs do or should do) | Health promotion, BCC, other advice | BEH | Provide advice or behaviour change communication, home visits for health promotion. Newborn advice frequently coded here. |
|  |  |  | Diagnostics | DX | Availability of diagnostic testing appropriate to services (i.e. 3 diseases). Differential diagnosis of fever, other diagnostic tools (thermometers, stethoscopes), and nonspecific mention of blood slide also coded here. Includes statements about testing before treating, which in some cases relate to QOC/PROTADH and may be dual coded. |
|  |  |  | Treatment | TX | Provide specific care or treatment, beyond availability of drugs. Absence of newborn care coded here, although treatment not part of the iCCM package (analysed by iCCM component.) |
|  |  |  | Follow up | FU | Provide follow up services post-care. Gaps in follow up services (i.e. newborn). Postnatal home visits. |
|  |  |  | Referral | REF | Refer to health centre (provide referral note and help facilitate). Includes mention of advising mothers to take newborn to health centre. NB: Some statements emphasize provider compliance (a QOC issue) with referral protocol (e.g. they immediately refer cases can't manage); these are also coded QOC/PROTADH. Issues related more to the organisation of referral (how they manage the referral process) are coded ADEQ/ORGSER. |
| Availability - Skilled Personnel | AVAILPER | Sufficient numbers of skilled personnel are available to perform services  (NB: quantity of personnel; perceived quality relates to acceptability) | Insufficient number or should increase number of VHTs | INSUF | Perceive insufficient number of VHTs to be available. Number of VHTs performing iCCM should increase. Often relates to poor availability of VHTs (ADEQ/HRS) or services (AVAILRX/SO). Also includes mention of desire of choice of service provider (relates to acceptability). NB: Non-iCCM VHT KII responses about wanting to provide iCCM treatment coded here (Sometimes related to insufficient coverage or poor iCCM VHT performance but not always). |
|  |  |  | Insufficient number (coverage) | INSUFCOV | Insufficient number of VHTs relates to large size of village or inadequate coverage (accessibility). Includes issues related to equity. |
|  |  |  | Insufficient number (demand) | INSUFDEM | Insufficient number of VHTs relates to increased population or demand for services (or anticipated growth in demand, population increase) |
|  |  |  | Number will increase in future | INCFUT | Expectation that number of iCCM VHTs will or should increase in the future |
|  |  |  | Sufficient number of VHTs | SUF | Two VHTs performing iCCM is enough |
| Availability - Drugs and commodities | AVAILRX | Availability of iCCM treatment and commodities *[NB: perceived quality or effectiveness of treatment offered is coded under acceptability]* | Stockout | SO | Inconsistent availability of treatment that is part of iCCM package (Coartem, amoxicillin, etc) or other statement that 'drugs are not enough', insufficient quantities to meet demand. |
|  |  |  | Available | AV | Drugs are available or provided. Includes a few mentions of overstocking, drugs returned to health centre to avoid expiration. |
|  |  |  | Complete dose provided | FULDOS | A 'full' or complete dose is provided. (Code split from 'drug availability', as frequently emphasized. Relates to perceptions of quality of care and differentiates from other HSPs where patients purchase quantity that can afford.) |
|  |  |  | Other drugs | OTHRX | (Lack of) treatment availability for three diseases that is not provided as part of iCCM package but pertinent to three diseases or newborn care and perceived to be important for U5s (e.g. injections, drips, panadol, alternative drugs for 3 diseases, etc). Also coded here that don't have newborn dosage. |
|  |  |  | Commodities | COM | Availability of commodities for iCCM service provision (gloves, buckets, cups, jerricans, clean water, protective gear, scale). Primarily related to drug provision or clinical practice. NB: referral forms coded as ADEQ/ORGSER |
| Availability - Other Services | AVAILOTH |  | Other (non-iCCM) services, treatment or diagnostics | OTHSER | (Lack of) availability of non-iCCM package services, treatment or diagnostics perceived to be important (e.g. immunization, family planning, HIV, ANC, delivery, dental services, other illnesses, mosquito nets, typhoid diagnostics, TB) |
|  |  |  | Services/drugs at referral HC | REFHC | Service and drug availability at the referral health centre |
|  |  |  | Services for other populations | OTHPOP | Lack of service availability for non-targeted populations (older children and adults).  Coded as availability ('do the offered products and services correspond with the needs of poor people?') NB: in a few VHT reports, this emerges as an issue of acceptability (caregivers get upset when say can't treat over-fives). Coded here in order to group all statements related to other populations. |
| **Accessibility** |  | **Location of supply is in line with the location of the community** | | | |
| Accessibility - iCCM | ACCES | Accessibility of iCCM services via VHT | Proximity / Distance | PROX PROXEQ | Accessibility relates to distance from VHT. VHT location near/far, VHTs located 'within the village' coded here. Sometimes mention equity (captured as subcode 'PROXEQ') |
|  |  |  | Coverage | COV  COVEQ | Accessibility relates to location of VHTs (given size of catchment area and ability of VHT to cover). May be sufficient number, but not located in right areas or distributed appropriately across village, limiting geographical access. Often relate to equity (captured as subcode 'COVEQ') NB: Differentiated from issues related to availability of services (i.e. insufficient number of VHTs/personnel inadequate to meet demand due to large coverage area). |
|  |  |  | Ease of access | EASE EASEEQ | Statement mentions ease of access, without further specificity, or difficulty to access and/or locate. Also may focus on time to access (can access at night, can access immediately) as a consequence of location. NB: Statements reporting an impact, ('we can easily access treatment') are coded TXACCESS. |
|  |  |  | Security | SEC | Issues related to insecurity (primarily associated with accessing at night) |
|  |  |  | Transport (for care-seeking) | TRANS | Need or lack of need for transport to access services at VHT. (Not often raised as a separate issue, as usually raised in relation to PROX (can just walk there) or when transport mentioned, usually mentioned as an affordability issue (AFFORIND/TRANS). |
|  |  |  | VHT access via phone | PHONE | VHT accessibility via phone |
|  |  |  | VHT proximity | PROXVHT | Proximity for provision of services. Because VHTs close to client, easy to provide services (e.g. follow-up) |
|  |  |  | VHT Transport | TRANSVHT | Accessibility for provision of services. VHTs require transport to conduct follow-up, collect drugs from health centre, etc. Also includes rain gear (gumboots, umbrellas, jackets), as these commodities are perceived as necessary for accessing households via foot to conduct follow-up, etc. Does not include comments related to VHT-provided transport to facilitate referral (ACCESREF) |
| Accessibility - Referral | ACCESREF | Accessibility of referral made by VHT | Proximity / Distance | PROX | Accessibility of referral health centre relates to distance |
|  |  |  | Referral Transport | TRANSREF | Ease or difficulty of obtaining/locating transport for referral (independent of cost). Includes comments related to VHTs having transport that could facilitate referral. |
| **Affordability** |  | **Prices of services (direct and indirect) fit the client's income and ability to pay** | | | |
| Affordability (iCCM services) Direct Costs | AFFORDIR | (Presence or lack of) direct costs associated with accessing iCCM services via VHT | Direct Cost - Free | FREE | Free services, no payment required. NB: impact statements related to avoidance of fee-for-service payments or money saved are coded as FCIMP. |
|  |  |  | Cost to VHT | COSTVHT | Costs related to service provision incurred by VHT. Any kind of financial or material cost (for transport to restock drugs, drinking water, paper, pens, etc) associated with service provision. |
| Affordability (iCCM services) Indirect Costs | AFFORIND | (Presence or lack of) indirect costs associated with affordability of accessing iCCM services | Transport | TRANS | Cost of transport to access iCCM services (not including referral) |
|  |  |  | Time (plus livelihood impact) | TIME | Time lost or gained due to accessing services. Includes comments about wait times, time lost or gained for other activities/work. Often reported as impact (e.g. no longer losing time waiting in queues at health centre has a livelihood impact). A few statements also coded here that do not explicitly mention time, but focus on implicit value of time saved. |
|  |  |  | Travel | TRAV | Opportunity costs associated with traveling long distances (not specific on time, cost, security etc) Often reported as impact. |
|  |  |  | VHT Time | TIMEVHT | Time lost or gained due to providing services. Often reported as impact (livelihood impact) |
|  |  |  | VHT Travel | TRAVVHT | Opportunity costs to service provider associated with traveling long distances (not specific on time, cost, security etc) |
| Affordability - Referral | AFFORREF | Affordability of referral made by VHT  *(includes referral for newborn care)* | Expenditure (direct cost or non-specified) | EXP | Direct costs of care at referral health centre (may be due to inadequate service availability, stockouts/ need to purchase drugs elsewhere), other non-specified expense related to referral or mixed types of expenditures. Often reported as impact. |
|  |  |  | Indirect cost | TRANSREF | Cost of transport to access referral. Often reported as impact. |
| **Adequacy** |  | **Organisation of iCCM meets community expectations** (i.e. how services are provided) | | | |
| Adequacy | ADEQ |  | Space | SPC | Adequacy of space allotted for services (VHT home) and set-up (resting bed, blanket, chair, mosquito net) |
|  |  |  | Clean and well-kept | CLN | Cleanliness of location of services (VHT home). Includes request for aprons. |
|  |  |  | Organisation of resources | ORG | Organisation of relevant materials (records, drugs, other) at location of services (VHT home). Includes materials needed for organization (drug storage boxes, etc) |
|  |  |  | Organisation of services | ORGSER | Organisation of service provision. Mostly related to referral (use of referral and counter referral notes, inadequate availability of referral forms, liaison with referral facility via phone, other VHT facilitation of referral (escort to referral HC), preferential treatment at HC when referred by VHT. NB: Caregivers frequently report this as an impact. NB: issue reported of having to see VHT before being able to use service at HC (must have referral letter or get sent back) mentioned as an acceptability issue |
|  |  |  | Supervision/monitoring of VHT performance | SUP | Adequacy of supervision of iCCM services. |
|  |  |  | Opening Hours | HRS | Appropriateness and adequacy of opening hours for VHT services. Includes references to both 'VHT absence' and 'VHT presence". NB: Sometimes overlaps with AVAILPER, e.g. if there were more VHTs, then there would be someone else to cover while 1 VHT is away. Also associated with some mention of 'lost time' (indirect cost) due to VHT absence. |
|  |  |  | Signposts to enable location identification | SIGN | Mention of need for signposts. Also relates to ACCESS (increasing awareness of location, being able to identify location of services). |
|  |  |  | VHT Compensation | SALVHT | Perceived adequacy of VHT compensation. Mention of provision of salary, allowance or other monetary compensation. Frequently mentioned in relation to different issues, e.g. if they are salaried, they will be more available or perform better. Also mentioned as an opportunity cost to VHTs and motivation. Includes mention of requests for livelihood generating activities for VHTs and risk of VHT attrition due to lack of salary. |
| **Acceptability** |  | **Characteristics of iCCM providers match with the expectations of the community** | | | |
| Acceptability | ACPT | Non-specific acceptability of VHTs performing iCCM services | Well-known; awareness | AWR | Awareness (or need for greater awareness) of VHTs and services provided by VHTs. VHT are well-known and recognised by community. |
|  |  |  | Low knowledge or awareness | LOWAWR | Low awareness or knowledge of VHTs or iCCM services. |
|  |  |  | iCCM users (accepters) | USE | Characteristics of persons who accept, utilise or most appreciate iCCM services. To consider when analyse utilisation. |
|  |  |  | iCCM nonusers | NONUSE | Characteristics of persons who don't accept, use, or benefit from VHT services, e.g. don't trust services, prefer to use traditional healer first, or seek to 'minimize' VHTs. Includes a few mentions of non-timely-use (barriers to timely treatment seeking). To consider when analyse utilisation. |
|  |  |  | VHT uniform | UNIF | Need for VHT uniforms. Most often relates to perceptions of VHT legitimacy or acceptability (to avoid minimizing, increase status or awareness, improve people's perceptions of and acceptability of VHTs). In a few instances appears to be more about identifying location of services or an adequacy/cleanliness issue, but nonetheless captured here in one place. |
|  |  |  | VHT volunteerism | VOLVHT | VHT acceptance of providing volunteer service. Agreement to work as volunteer. Caregiver acknowledgement that voluntary work. |
| Acceptability - VHT Characteristic or personality | ACPTCHAR | Characteristics or personality of VHT influences acceptability | Dedicated | DED | VHT acceptance related to perceived dedication or commitment to service provision (including personal sacrifice). |
|  |  |  | From the village | FRMVIL | VHT acceptance/trust related to being from the village ('one of us') or to being selected by village. |
|  |  |  | Attitudinal characteristics | ATD | Welcoming and caring. VHTs make caregivers/children feel welcomed and cared for. VHT acceptance related to perceptions of how handled by VHT (treats calmly, kind). |
|  |  |  | Performance characteristics | PERF | VHT acceptance or trust related to perceived willingness to serve (always ready to serve, activeness, volunteerism); activeness in follow-up; responsiveness (immediately, fast), 'admit mistakes'; demonstrate by example. Poor performance, low motivation, do not prioritise iCCM work (PERFPR). |
|  |  |  | Integrity | INTG | VHTs act with integrity, treat patients fairly, provide services equitably, are non-discriminatory, don't sell resources/drugs, etc. Involvement in politics. |
|  |  |  | Gender | GEND | Gender-related issues affecting acceptability |
| Acceptability - Provider Competence | ACPTCOMP | VHT acceptance or trust related to perceptions of VHT competency or skills | Confidence in competency / ability to treat | CONF | General expression of confidence in VHT ability to effectively treat. General expression of trusting the VHT based on perceived competency or skills. Can be a perceived result of something else, e.g. because they are clean and well-organised, we feel confident in quality of service. |
|  |  |  | Qualification, skill level | QUAL | VHT skill level or qualifications meet community expectations. Acceptability relates to perceived competency of VHT. Are they qualified to provide the services they offer? Perceptions of adequacy of training and VHT knowledge. Includes general statements about need for more training or skills (where not about service availability) or supervision. |
| Acceptability - Treatment & Explanation | ACPTTX | Acceptability of provided / available treatment and of provider communications around treatment plan | Effectiveness | EFFECT | Perceived quality or effectiveness of treatment offered. Includes concerns about expiry. |
|  |  |  | Dosage | DOS | Perceived ease of administration, side effects, acceptability of dose to child, appropriateness of paediatric formulation. Includes comments on avoiding injections, herbs, harmful treatments. |
|  |  |  | Explanation | EXPL | Provider explanations (and other communications) meet expectations. Perceived appropriateness of provider explanations, information and follow-up home visits. |
|  |  |  | Referral | REF | Acceptability of referral provided by VHT. |
|  |  |  | Absence of treatment | ABS | Acceptability of not receiving treatment (e.g. present with cough, but no fast breathing) |
|  |  |  | Avoid death in hands of VHT or at village level | DTHVHT | Relates to immediate referral (as course of care): VHTs refer immediately, ensuring child does not die under their care, that they are not blamed for death or problem with child. Reflects acceptability of referral services. |
| Acceptability - VHT Selection and Community Engagement | ACPTSEL  COMENG | Acceptance of VHT selection process (may or may not impact acceptability of VHT).  Community involvement in VHT selection, location, monitoring, reporting (NB: there was overlap between these two categories. Analysed together.) | VHT selection | ACPTSEL | Appropriateness of selection process, criteria, outcomes |
|  |  |  | Community involved | INV | Community involved in selection process (e.g. meetings held), location, monitoring, reporting. |
|  |  |  | Awareness of community role in iCCM | AWRINV | Community not aware that should be involved in selection, monitoring, reporting, etc. |
|  |  |  | Community not involved | LOWINV | No or low level of community involvement |
| Acceptability - VHT recognition and motivation | ACPTMOT | Relates to morale of service provider (acceptability of service provision to VHT) | Verbal feedback (thank you) | THK | Provide verbal commendation or appreciation: say thank you, provide positive feedback, recommend to others, speak well of them in the community, pray for them. |
|  |  |  | Utilise services (as a form of motivation) | UTIL | Utilise the services offered by VHTs or encourage others to utilise services |
|  |  |  | In kind | INKIND | Provide in kind support to VHT (provide food, labour) |
|  |  |  | Belief that VHT already compensated or that it is their obligation | VHTPAID | Don't motivate due to belief that VHT is already compensated/paid or that it is their obligation to provide services |
|  |  |  | Otherwise do not motivate | NOMOT | Do not provide any motivation |
| Acceptability - Patient/ Community (to VHT) | ACPTPT | Patient behaviour meets provider expectations | Patient cleanliness | CLN | Patients dirty VHT homes; caregivers do not present with clean children. |
|  |  |  | Patient mistreatment of VHT | RUDE | Caregiver insults or is rude to VHT |
|  |  |  | Other caregiver behaviour | CGBEH | Poor utilisation of services, etc. Mostly captured under other recommendations (as statement usually about what caregivers need to do). Here capture statements of the 'problem' |
| **Perceived Impact** |  | **Perceived effects of iCCM on health service delivery and children under five** | | | |
| Treatment Access | TXACCESS | General statement about ability to access treatment as result of iCCM (reported as impact) - essentially a summary of 5 dimensions | Timely treatment | TIMTX | Able to access treatment in time due to iCCM services. Often reported as a result of something else, e.g. because services in village, free, drugs available, etc |
|  |  |  | Able to access treatment | YES | General statement about being able to access treatment as an impact of iCCM services. Usually reported as a consequence of or in association with something else (dual coded). |
|  |  |  | Not able to access treatment | NO | General statement about not being able to access treatment or timely treatment as a result of some aspect of iCCM (stockouts, provider performance, etc) |
|  |  |  | Accessing services at referral HC | REF | Able to access treatment (promptly) at referral facility. Often impact of ADEQ/ORGSER and dual coded. |
| Utilisation | UTIL | Community use the (iCCM) services available  *[linked also to acceptability coding - who uses/doesn't use]* | General | GEN | General mention of utilisation of VHT iCCM services or treatment seeking at VHT (as an impact). |
|  |  |  | Timely treatment seeking | TIMTXSK | Specific mention of timely treatment seeking behaviour (as an impact of iCCM). Also includes rare mention of lack of timely treatment seeking. |
|  |  |  | Return use | RTN | Continued or return utilisation of iCCM services. (Usually a secondary code, e.g. acceptability, adequacy, follow-up, patient satisfaction with services promote return utilisation) |
|  |  |  | Change in utilisation following iCCM | CHN | Explicit statement about utilisation trends following iCCM implementation, e.g. we no longer go to private clinics now that we seek care at the VHT). |
|  |  |  | Other health service providers | OTHHSP | Circumstances when need to use other HSPs rather than VHTs or groups of people who remained using other HSPs. |
| Quality of care | QOC | Effect of iCCM on patient treatment adherence, perceived provider compliance with iCCM protocols (NB: Other items related to QOC are coded under relevant dimension of access: provider qualifications are coded ACPTCOMP/ QUAL; lack of diagnostics or use of diagnostics (test before treat) coded AVAIL/DX; perceived quality/safety of drugs coded ACPTTX/EFFECT). Some data also relate to improved patient safety (drug quality/ acceptability, expiry, etc.) as well as avoiding dangerous treatments (at private providers); caregivers report these as health impacts. | Treatment adherence | TXADH | Impact on treatment adherence. Often reported as an impact of VHT follow-up. |
|  |  |  | Identify other health problems or gaps | MONT | Impact on obtaining other health-related information or issues to be addressed through monitoring. Impact on surveillance and incidence reporting. Identification of health problems in community to be addressed. Reported as an impact of VHT follow-up. |
|  |  |  | Treatment plan decision making - the 'right treatment' | RTTX | Impact of iCCM on determining correct treatment plan, ensuring 'correct dose' is prescribed. NB: comments about receiving a 'full dose' coded under AVAILRX / FULDOS. |
|  |  |  | Provider adherence to key iCCM protocols | PROTADH | Mention of following sick child job aide, immediate referral. Most of the time not reported as an impact, but rather just what providers do. Applied as secondary code and first coded under appropriate dimension, e.g. AVAILSER/REF |
| Consumption of health services | HSCONS | Impact on consumption of health services | n/a | n/a | Reduced use of health facility services; reduced hoarding of medicine for future use |
| Health status | HLTHSTAT | Effect of iCCM on health status of the community *Impact on health outcomes for children under five (and broader community if mentioned), plus some other 'intermediate impacts' which affect health status* | Mortality reduction | MORTRED | Perception of decreased child deaths, saves lives. Zero child deaths since iCCM. |
|  |  |  | Mortality OR condition worsens | MORT | Persistent mortality, no decrease in mortality, risk of mortality, no improvement in illness or worsening of condition. Often stated as the result of delayed treatment due to VHT absence or referral process. |
|  |  |  | Disease reduction | DISRED | Perception that incidence of particular illness has reduced. Includes avoiding disease spread, infecting other children. Includes rare negative perception that incidence of particular illness has not reduced or is still persistent, e.g. untreated children reinfecting treated children). |
|  |  |  | Healthy kids | HLTHKID | Perception that children are healthy as result of iCCM, experience correct child growth and development. Often stated with knock-on benefits (increased human capital). Includes mention of improved nutrition. |
|  |  |  | Cure | CUR | Empiric statement about successful treatment experience. Also partially successful or mixed experiences (‘most of the time they get better’). |
|  |  |  | Avoid severe illness | SEV | Avoid more severe illness, admissions and associated risks, costs, trauma, sequelae (because treated early) |
|  |  |  | Hygiene, sanitation, environmental management | SANIT | Impact on village and household environment. Improved hygiene practices, sanitation, cleanliness. Mention of 'model village'. Includes environmental management, e.g. reduction of breeding sites. |
|  |  |  | Other healthy behaviour change | CGBEH | Impact on other caregiver healthy behaviours as a result of VHT BCC. |
|  |  |  | Population increase | POP | Increase in population, fertility, birth rates, perception of increase in reproduction because of access to healthcare. (Technically, a demographic change rather than health status.) |
|  |  |  | Other | OTH | Uncategorised impacts on health status. Avoid risk of accidents during transport for accessing health services. |
|  |  |  | Health risks to VHT | RISKVHT | Perceived health risks posed to VHT or VHT's household by providing services, e.g. increased exposure to disease. |
|  |  |  | Health benefits to VHT's children | BENVHT | Perceived health benefits to VHT or members of VHT's household by providing services, e.g. also access medicine. |
| Patient (caregiver) satisfaction | PTSAT | Patient / community satisfied with iCCM services | Satisfied | SAT | General statement reflecting satisfaction. |
|  |  |  | Cure or symptom relief | CUR | Empiric statement about satisfaction due to effective treatment. Some overlap with HLTHSTAT. |
|  |  |  | Reassurance | REASS | Impact of iCCM on caregiver peace of mind. Caregiver reassured by VHT / iCCM service provision/availability, reports enhanced sense of wellbeing, no longer worry |
|  |  |  | Cost | COST | Patient satisfaction primarily related to cost |
|  |  |  | Not satisfied | UNSAT | Reports reflecting caregiver dissatisfaction with iCCM implementation |
|  |  |  | Community / family satisfaction | FAM | Reports about overall increased well-being of family, household or community |
| VHT Satisfaction | VHTSAT | VHT satisfied with iCCM service provision experience | Satisfied | SAT | Statement reflecting general satisfaction; positive effect on morale |
|  |  |  | Personal growth or change | PERS | Statement reflecting on personal growth or changes as a result of iCCM. NB: some overlap with HCIMP/VHTK code. |
|  |  |  | Unsatisfied | UNSAT | Statement about reasons why unsatisfied; impact on low VHT morale |
| Equity Impact | EQIMP | Equal access to iCCM services by those in equal need |  |  | Reported impact on equity |
| Social capital impact | SCIMP | Perceived impact of iCCM on social capital (social networks and affiliations) | VHT social capital / status | SOCSTAT | Impact on VHT social status (social recognition, reputation, power, influence) or social capital and use of this social capital. Includes statements reflecting changes in VHT position in society; community members seek out VHT as person of authority, source of wisdom. Also serves as evidence of ACPT / popularity of VHTs.) |
|  |  |  | VHT-Community relationship | VHTCOM | Impacts on relationship between the VHT and the community. Also statements reflecting community acceptance of VHT through support provided, related to community engagement. |
| Financial capital impact | FCIMP | Perceived impact of iCCM on financial capital (cash and credit) | Resource allocation (household spending) | HHSPEND | Impact on household finances and resource allocation. Cash can be allocated for other needs (school, food) due to decreased health expenditures. *NB: some of these have explicit knock-on health benefits (e.g. spend money on more nutritious food for children or on medicine for older children)* |
|  |  |  | Money saved | SAVE | Money saved due to decreased health expenditures (no mention of impact on resource allocation) OR mention that 'no longer spend' / 'reduced health expenditures'. Includes rare mentions of no longer needing to borrow money for health expenditures, reduced debts or no longer needing to sell assets for health expenditures. |
|  |  |  | Expenditure | EXP | Health expenditure required due to gap or limitation of iCCM service provision. (In case of stockouts, lack of newborn care available as part of iCCM, referral [where perceive VHT should be able to provide service instead of referring].) Includes rare mentions of needing to sell assets or borrow for health expenditures. |
| Human capital impact | HCIMP | Perceived impact of iCCM on local knowledge, education and skills (including health-related skills and knowledge) | Education | EDU | Increased school attendance (because children healthy) |
|  |  |  | Caregiver knowledge | CGK | Impacts on caregiver knowledge, e.g. how iCCM has helped caregivers to better care for children, make decisions about child health, etc.) Mentioned frequently in relation to follow-up services provided by VHTs. NB: behavioural changes, even where knowledge implied, are coded as HLTHSTAT. |
|  |  |  | VHT Knowledge | VHTK | Changes in VHT knowledge or skills as a result of iCCM. (VHTs were asked about impact of skills on self.) |
| Other Impact | OTHIMP | Means of ensuring that all perceived impacts are captured | Domestic violence | DOMVIOL | Reduction in domestic violence mentioned as impact of iCCM |
|  |  |  |  |  |  |
