# Supplementary material for: Integrated community case management in a peri-urban setting: a qualitative evaluation in Wakiso District, Uganda
Source: BMC Health Serv Res. 2017 Nov 28;17:785. doi: 10.1186/s12913-017-2723-0 (PMC5706411; doi:10.1186/s12913-017-2723-0)
Supplement: Supplementary file 2 — Summary of user and VHT member perceptions of iCCM implementation in peri-urban settings. A preliminary thematic summary was prepared for each of the evaluation criteria, in alignment with the ‘Health Access Livelihood Framework’. (DOCX 40 kb) [file 12913_2017_2723_MOESM2_ESM.docx]

**Annex 2.** Summary of user and VHT member perceptions of iCCM implementation in peri-urban settings

| Evaluation criteria | Value of iCCM services | Gaps potentially requiring further attention | Factors influencing choice of service provider |
| --- | --- | --- | --- |
| Availability | Services for malaria, pneumonia and diarrhoea treatment match communities’ needs  Some diagnostics used (timers)  Conduct follow-up visits (but gaps in regularity or coverage in two villages) | Need for more diagnostic tools (malaria RDTs, better quality timers for fast breathing), newborn care (but some VHT members wary of treating newborns)  Users recommend more iCCM VHT members (linked to VHT member distribution and resource gaps); some recommend training non-iCCM VHT members (improve coverage, availability, equity and lessen workload)  Poor drug availability a top problem | Follow-up services enhance VHT acceptability and utilisation  *Limited types of service (the ‘three diseases’)*  *Drug stockouts necessitate use of other service providers*  *Absence of RDTs and other diagnostics prompts use of other health service providers (both initial care seeking and via referral)* |
| Accessibility | VHT members proximally located and accessible, easily reached by foot | Inequitable coverage linked to low community engagement in VHT selection process (in highly peri-urban villages) |  |
| Affordability | Free of formal and informal charges (no out-of-pocket costs)  Caregivers avoid indirect costs (time, travel for health-seeking); ‘saved time’ allows caregivers to engage in productive activities | Some time wasted due to stockouts, limited equipment and VHT member absence | High cost of medicine (ACTs in particular) in private drug shops  Significant time travelling to and waiting at health facilities (sometimes recurrent, as drugs not available); time seeking herbal remedies |
| Adequacy | Long hours of service availability, even at night; caregivers acknowledge voluntary work and that VHT members have other responsibilities too  Clean services, good hygiene practices, well-kept drugs, resources and records in most villages; engenders confidence in VHT services and medicine safety; encourages return use. | Occasional VHT member absence (in one village, a top problem due to VHT member’s full time employment outside the home)  Some difficulties in maintaining cleanliness due to lack of facilities and supplies  Inadequate space for care provision (separate treatment area recommended)  Need larger medicine boxes and storage containers; seating for waiting and consultation; beds for treatment and admissions; and light for night-time care provision | *VHT member absence leads to utilisation of private clinics or health facilities* |
| Acceptability | Majority of community aware of iCCM services  Community engagement mixed; high involvement attributed to active local council chairman, effective sensitization, community receptiveness to programme), enhancing VHT acceptability and accessibility (location)  High acceptability of VHT characteristics: attitudes (welcoming, caring, ‘perform jobs with love’); performance (efficient, dedicated, perform with integrity, equitable); and competency (trained, qualified) – confidence increased over time and attributed to effective treatment, referral and follow-up services, following job aide and trust of a known person  High treatment acceptability (effective, fast-acting, good quality, unexpired, no side effects); wide acceptance of dosages and formulation | Inadequate involvement in VHT selection in most peri-urban villages (poor awareness of program initiation, lacked opportunity), contributed to poor coverage (location), ownership and politicisation of services (in one village)  Less involvement and transparency in second stage of selection (for iCCM service provision)  Less awareness, trust of VHT member qualifications and acceptability by wealthy community members (not directly targeted by programme)  Poor acceptability of absence of services for older children  Request further VHT training and competencies  Recommend certificates of training  Limited mention of persons distrusting iCCM medicines (believing politically motivated)  A few mixed reports on ORS acceptability and pneumonia treatment effectiveness | VHT member attitudes attract patients: ‘care’, do not abuse or shout at caregivers (unlike HFWs, private clinics)  Not ‘business-oriented’ like private clinics; 'do not insist’ on treating illnesses cannot manage  Prefer VHT medicines to some treatment at private clinics and health facilities (perceived to have adverse effects or be ineffective); oral administration preferred to injections |
| Referral  *(availability, accessibility, affordability, adequacy, acceptability)* | VHT members refer appropriately when unable to manage illness  Effective service organisation, caregivers benefit from referral letter, prioritised care at health facility  Acceptability increased over time (appreciate VHT limitations, that referral sometimes necessary); referral not only potentially life-saving, but essential for upholding VHT members’ reputations | Lack of transport a top problem; VHT members sometimes incur costs to ensure referral completion  Transport difficult and expensive  Gaps in service availability and out-of-pocket costs at referral health facility:  Sometimes sent back or 'forced’ to first obtain referral from a VHT member  Gaps in post-referral service organisation (no counter-referral); referral facility services (health workers unavailable at night, stockouts at referral facilities leading to referral elsewhere); and inadequate supply and low acceptability of the referral form | Effective referral services distinguished VHT members from other healthcare providers  VHT members were responsible, referred immediately and 'did not insist’ on treating illnesses they could not manage, with caregivers in two villages contrasting this with clinic services. |
